# "It depends on what you mean": a qualitative study of Swedish health professionals' views on health and health promotion

**DOI:** 10.1186/1472-6963-9-191

**Published:** 2009-10-21

**Authors:** Helene Johansson, Lars Weinehall, Maria Emmelin

**Affiliations:** 1 Epidemiology and Public Health Sciences, Umeå International School of Public Health, Public Health and Clinical Medicine, Umeå University, SE- 901 85 Umeå, Sweden; 2 Swedish National Institute of Public Health, SE- 831 40 Östersund, Sweden

## Abstract

**Background:**

The role of health services must be re-oriented towards health promotion to more effectively contribute to population health. One of the objectives of the Swedish public health policy is that health promotion and disease prevention should be an integral part of the health care system and an important component of all care and treatment. However, the uncertainty about what the concepts of health and health promotion mean poses a challenge for implementation. Depending on how these concepts are interpreted, the attitudes of health professionals toward health promoting practices will differ. Thus, a more in-depth understanding of health professionals' views can be a starting point for a discussion about the values and attitudes that influence the current health care system and about the barriers and possibilities for future development of a health promoting health service.

**Methods:**

Seven focus group discussions (n = 34) were carried out with health professionals, from different health care settings, to understand how they communicate about health and health promotion. The data were analyzed using qualitative content analysis.

**Results:**

The analysis of health professional's general understanding of the concept of health resulted in the category; *a multi-facetted concept*, whilst the category; *a subjective assessment *describes what health means to themselves. A third category; *health is about life, the whole life*. describes their understanding of health as an outcome of a multiplicity of contextually dependent determinants.

The health professional's multiple ways of associating health promotion to disease prevention suggest a concept that is diffuse, elusive and difficult to apply in practice. Despite a shared view of health, the health professionals described their health promotion role very differently depending partly on how the concept of health promotion was interpreted. The analysis resulted in the development of three ideal types, labelled *the demarcater*, *the integrater *and *the promoter *describing different strategies for handling a health promotion role in practice

**Conclusion:**

The study suggests that different interpretations of what constitutes health promotion can lead to unnecessary misunderstandings and pose barriers to further development of a health promoting practice.

## Background

During the last three decades, it has been suggested that the role of health services must be re-oriented toward a health promoting direction in order to more effectively contribute to population health. The World Health Organization (WHO) has been an important source of inspiration in this respect and a number of documents has supported their ambition [1-3]

In 2003 when the Swedish Parliament (Riksdag) adopted new national public health objectives, the health sector was considered as one of the most important arenas for public health activities. Due to its specific competence, broad knowledge, authority and wide-scale contact with the population, health service is of considerable importance for long-term health development. Health and medical care that more actively promote good health was therefore given its own objective. It stated that health promotion and disease prevention perspectives should be an integral part of the whole health care system and a palpable component of all care and treatment [4]. The public health policy was updated in 2008 [5].

Promoting health and not just preventing disease and injury may in several ways imply a new undertaking, which causes uncertainty regarding the implications for practice. Depending on how the concept of health is interpreted, the attitudes of health professionals toward health promotion will differ and this interpretation will also influence the goal orientation of different types of health care services [6]. The frame of health promoting efforts may also vary, depending on which health determinants are targeted [7].

Clarity is a prerequisite for successful public health work. Well thought-out and shared views of the concepts of health and health promotion become vital for the prospects of developing and running a health promoting practice.

### Defining health

Health is a familiar term or phenomenon in everyday life, representing a universal experience that everyone knows about [8]. But if one looks beyond what is taken for granted, health as a concept is complex and can be viewed from numerous perspectives, each with different definitions [9]. Arnold and Janssen Breen [10] portray ten images of health; i. e., the antithesis of disease, a balanced state, growth, a functional capacity, goodness or fit, wholeness, well-being, transcendence, empowerment and health as a resource, which all together reveal the complexity of the concept.

A more simplified picture of the extensive literature on health allows a discussion based on two main perspectives. The first is called the (bio) medical or the biostatical perspective of health. This perspective contains theories focusing on the body, viewing health and disease as the opposites of each other and focusing on the disease aspects. According to this perspective, a healthy person is one who does not have any disease or maladies [6,11]. The second perspective is more socially oriented, often called holistic, indicating that health and disease are concepts related to the whole human being, where the social context has to be taken into account [11]. This means that there are other criteria to health than absence of disease, such as well-being, happiness, human flourishing, and ability to realize vital goals or possibilities to function as a whole [12].

The WHO definition of health from 1946, which described health as "a complete state of physical, mental and social well-being and not merely the absence of disease or infirmity", is probably the most well-known and cited one. In those days this definition was seen as innovative because of its broader approach to health, but it has also over the years been criticized for being both unrealistic and idealistic [13]. WHO later revised the definition, and today health is considered as less of an abstract state. Health is defined as "a resource for everyday life, not the objective of living. It's a positive concept emphasizing social and personal resources, as well as physical capacities" [1].

Although health is one of the basic concepts within health care, we do not know so much about health professionals' perceptions of health. We have identified only three published Swedish studies in this field. Two of them [14,15] focused on occupational therapists' views on health, which showed that they most frequently hold a holistic view of health and to lesser extent a biomedical view. The third study focused on nurses within mental care, which indicated that the concept of health was not sufficiently defined [16].

### Defining health promotion

Like health, health promotion is a complex concept, frequently used, but often ill-defined. The term, which dates back to the 1970's, has been ascribed different content in different contexts [17] and its meaning has changed over time [9,13,18]

The "Ottawa Charter" [1] considers health promotion as "the process of enabling people to increase control over, and to improve, their health. To reach a state of complete physical, mental and social well-being, an individual and group must be able to identify and to realize aspirations, to satisfy needs, and to change with the environment". This definition stresses that health promotion is a process that enables people to take control over their own health. The Ottawa Charter contributed by emphasizing the need for a new understanding of health promotion. The definition indicated an important shift from focusing on modification of individual risk factors or risk behaviors to addressing the "context and meaning" of health actions and the determinants that keep people healthy [19].

According to WHO [20] health promotion should represent a comprehensive social and political process that not only embraces actions directed at strengthening the skills and capabilities of individuals, but also actions directed toward changing social environmental and economic conditions so as to alleviate their impact on public and individual health.

However, according to a review by Medin & Alexandersson [6], there is still great confusion about what health promotion is. They show that health promotion can be described in terms of actions, states which can be cured, counteracted, helped or promoted, goals, processes or strategies on different structural levels. This diversity of ideas in turn depends on factors such as how health is defined, on what structural level health interventions will take place; individual or population level, and if prevention is considered to be part of health promotion or if the terms are attributed to separate dimensions. International studies focusing on nurses and general practitioners also indicate that the meaning given to health promotion is unclear [21-23].

The uncertainty about the concepts of health and health promotion is a challenge for the health sector, where health professionals with various training and experiences are supposed to work together based on a joint agreement about basic values and toward a common goal. Thus, a more in-depth understanding of how health professionals, themselves, view these concepts is important. This can be a starting point for discussion about the values and attitudes that influence the current health care system and about the barriers and possibilities for further development of health promoting activities within the health care system.

### Aims of the study

The overall objective of this study was to understand how health professionals communicate about health and health promotion with an emphasis on the implication for practice. The specific aims were to explore how they:

• interpret the concepts of health and health promotion

• perceive their role in health promoting practices

## Methods

### Study design

The study had a qualitative research design based on focus group discussions. This method of data collection utilizes the group dynamic and is well-suited for exploring people's individual views and experiences of concrete phenomena as well as of more abstract concepts [24].

### Study setting

The study was carried out within the County Council of Västerbotten, located in northern Sweden. The county council administers three hospitals and 35 primary health care centers and provides health and medical services for 255,000 inhabitants.

### Sampling of informants

In this study health professionals were regarded as a "group" since we did not, at this stage, aim to compare different professional groups but to capture the range of experiences and views among them. Efforts were therefore made to include men and women with various professions, and with working experiences from different health care settings in both urban and rural areas. Initially only doctors and nurses were included in the study but the emergent design allowed for adjusting the sampling criteria based on preliminary analysis performed during the data collection. This meant that occupational- and physiotherapists, assistant nurses, counselors and midwifes were identified as being important to be represented. The actual sampling was facilitated by three key informants working at the county council administration, who in collaboration with the research group, suggested participants to invite for the group discussions.

### Data collection

Seven focus group discussions with a total of 34 participants were carried out during 2003-2004. The professional groups were represented by 1 counselor, 2 occupational therapists, 2 physiotherapists, 4 assistant nurses, 5 midwives, 10 nurses and 10 physicians. Nine of the participants were men and they belonged to the groups of assistant nurses, nurses and physicians. The participants working experiences included primary health care (including child health services, maternal health care and home nursing) and different hospital settings; dementia care, emergency care, gynecology and obstetrics, intensive care, medicine, orthopedics, palliative care, psychiatry, rehabilitation and surgery.

The groups varied from 4 to 6 participants, with five groups being homogeneous and two mixed in terms of profession. The discussions lasted approximately two hours and were performed in settings situated within the three hospital premises. To create a relaxed and comfortable atmosphere for the discussions the participants were invited to lunch. This provided an opportunity for them to be introduced to each other, as well as to the aim of the study. Three members of the research team took turns in being the "moderator" or the "assisting moderator". The role of the "moderator" was to keep the discussions on track, to encourage the participants to talk and to allow everybody to be heard. The role of the "assisting moderator" was to listen, observe and to take care of the audio recording and the note taking. The "assisting moderator" also made certain that all topics were addressed and in the end made comments or, if necessary, asked supplementary questions.

A thematic guide was used that focused on five main areas, three of which are reported in this paper. These are: i) what is health, ii) what is health promotion and iii) views about their health promoting role in practice. To facilitate the discussion the participants were also asked to individually write down three wishes for future health promoting health services. Their written suggestions were then taken as a starting point for a more in-depth discussion in the group. Before ending the discussions the participants had the opportunity to make a final statement summarizing their own views.

The emerging preliminary analysis resulted in increasingly focused discussions and since the last two did not generate much new substantial information we felt that we had reached theoretical saturation.

### Data analysis

**Figure 1:**
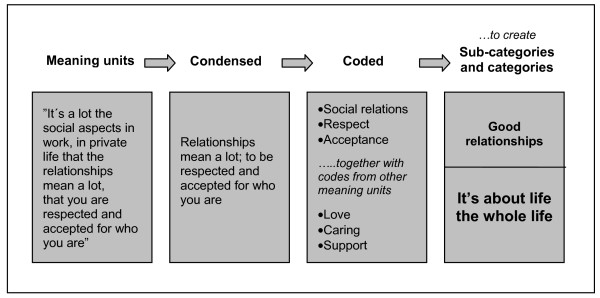
**Example of the coding process**.

The audio recordings were transcribed verbatim. The transcriptions were read through several times to get more familiar with the data. The analysis was performed using qualitative content analysis [25,26]. Texts dealing with the concept of health, the concept of health promotion and the health promotion role in practice were identified as meaning units that were condensed. The condensed meaning units were interpreted and labeled with codes. Codes with similar content were developed further into sub-categories and categories. The actual coding of the data was performed by the first author (HJ) but discussed and negotiated in regular "peer debriefing" sessions to enhance the trustworthiness of the research findings. Figure 1 illustrates the analysis process moving from meaning units to categories.

The first step in the analysis aimed at understanding the manifest meaning, i.e. the range and variation of attitudes and perceptions regarding the concepts of health and health promotion. Later when focusing more on health promotion in practice the analysis was deepened to include also the more latent meaning of the data. The emerging themes were developed by introducing Weber's concept of "ideal types" [27]. Originally these are theoretical constructs that metaphorically capture essential features of an investigated phenomenon, not necessarily found empirically. However, in our analysis the ideal types are grounded empirically even if they still can be seen as theoretical constructs in that one informant may contribute with characteristics attributed to several ideal types.

### Ethical considerations

The participants were informed in a letter about the aim of the research project and what participation in a focus group discussion meant. They were all informed that the participation was voluntary and that confidentiality would be secured throughout the research process. As an introduction to the discussions the participants were also encouraged to agree among themselves that the views shared would remain confidential. The study was approved by local Ethics Committee at the Faculty of Medicine, Umeå University (Um dnr 03-133).

## Results

The findings of our analysis are divided into three main areas relating to the concept of health, the concept of health promotion and health promotion in practice. Direct quotations from the discussions are included to illustrate how the interpretation is grounded in the data.

### The concept of health

The analysis of the health professionals' overall views about the concept of health resulted in two categories, "A multi-facetted concept" and "A subjective assessment", which are related to each other. It was also clear that the informants included several determinants for health in their definitions, i.e., those that are important both for maintaining and creating health. "Health is about life, the whole life" became the third category for the health professional's conceptual framing of health.

#### "A multi-faceted concept"

The health professionals' general understanding of the concept of health is that the meaning given to it differs. Apart from viewing health as being assigned a physical, mental and spiritual dimension, the tenor might vary among individuals, cultures and between different situations/contexts. The substance given to the concept is also believed to alter during the life span. The fact that the concept is multi-faceted is presumed to be a problem when health or health outcomes are measured and compared, because it is only the individual who knows what health means to him/her.

What is health? Well, it is about the body, the soul and the spirit?

Isn't it cultural, I mean that it will be defined differently depending on where in the world you are.

Health means different things depending of what phase in life you are in

#### "A subjective assessment"

In spite of the fact that the health professionals understand health as a multi-faceted concept, their statements regarding what health means to them are very similar to each other. They consider health as a subjective experience or feeling rather than an objective confirmable state. It is a matter of how one is doing (feeling) in the actual situation. Health, well-being and happiness are described as closely connected. Furthermore, health and disease are perceived as different dimensions and not as contradictions. This means that disease does not automatically lead to ill-health and that absence of disease is not a guarantee for health.

It is subjective. If you feel good you are healthy

It's about feeling good, being happy

You can feel healthy even if you are ill. It all depends on your own perceptions.

#### "Health is about life, the whole life"

**Figure 2:**
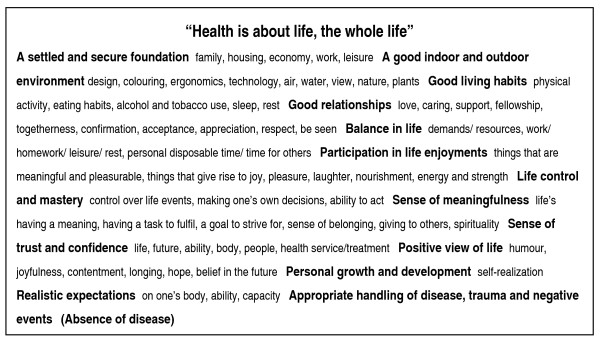
**A description of the category "Health is about life, the whole life" with the associated subcategories (bold) and codes (normal)**.

Figure 2 illustrates that the health professionals understand health as an outcome of a multiplicity of determinants, both individual and environmentally oriented. It is a matter of the whole - the individual must be considered in his/her entirety. Describing which factors contribute to health is not easy, because they comprise the whole life. Health is created in a continuous process, in the context of everyday life - at home, at work, in school and in leisure time, where physical, psychological (mental, emotional, existential), cultural and social factors interact. Individual factors were given plenty of space; factors that make them feel well and felt worth striving for. A number of factors are directly or indirectly linked to positive experiences, thoughts and feelings that give rise to nourishment, strength, energy and a sense of well-being. The sub-categories *realistic expectations *and *trust/confidence *derive from work experiences. Unrealistic expectations on one's life, body and capacity as well as of what health services can accomplish are, together with feelings of insecurity and lack of confidence, considered as threats to health, especially when it comes to young people. In Figure 2, the sub-category *"absence of disease*" is put within brackets, because there is no direct link between absence of disease and health. Nevertheless, absence of disease increases the possibilities for the individual to handle his/her own life and fulfill or satisfy his/her own needs, and thus it is considered as a determinant for health. Paradoxically, illness and negative events can act as a catalyst to health or increased health, through a contribution to personal growth and development.

The individual's own responsibility is emphasized even though health professionals acknowledge the responsibility for health by employers and public sectors in society. In general they see the individual as the only one who knows what influences his/her health and thereby is able to affect his/her situation.

### The concept of health promotion

The health professionals' descriptions of health promotion indicate a concept that stands out as diffuse and elusive. It comes close to an **all-embracing concept **that includes all actions, activities and strategies that directly or indirectly promote the health (sense of well-being) of an individual in both a short and long-term perspective. The measures can take place on different structural levels. Their definition also includes a "setting approach", meaning that the physical and social settings in themselves also have an impact on health.

Health promotion is also described as an "attitudinal approach" in the meeting with patients and relatives. It is not just about **what **to do or say, it is also a matter of **how**. Essential concepts linked to this attitudinal approach are a holistic view of humankind, participation, commitment and devotion, trust, concern, confirmation, encouragement, support and empowerment.

When health promotion was related to prevention we could differentiate between three approaches where: 1) health promotion and prevention were seen as separate concepts; 2) health promotion was seen as an extensive concept which included prevention; and 3) health promotion was seen as a companion to primary prevention.

### Health promotion in practice

**Figure 3:**
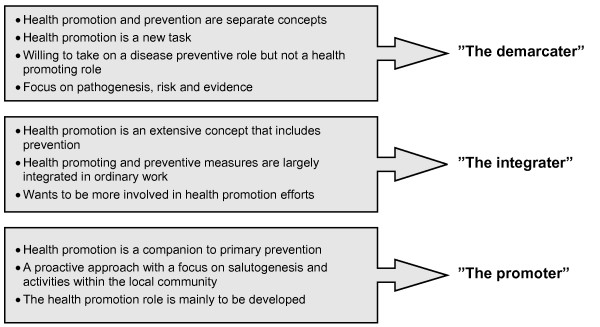
**The three ideal types and their main characteristics**.

The findings that it is possible to distinguish between three approaches to health promotion also emerged as important for understanding the health care professional's perceptions about and strategies for handling their health promotion role in practice. Their strategies are described as ideal types, which are labeled the *demarcater*, *the integrater *and the *promoter*. The main characteristics of each ideal type are given in Figure 3.

#### "The demarcater"

The advocates for this approach **distinguish between health promotion and disease prevention efforts. **Due to the fact that the main determinants of health reside outside the health sector's field of knowledge and are difficult to influence, the significance and the role of the health services as a public health promoter are questioned.

I belong to those who want to place this [idea] with health actually outside health care, because a great deal of it has to do with things that I believe lay outside our, both competence and possibility to affect, meaning all the social aspects, relationships and such

The demarcater is unwilling to take on a health promotion role. The main reason is that he/she neither considers himself/herself to be trained in fostering people's well-being or happiness nor perceive it as a duty. Additionally, the demarcater seems to believe that undertaking a health promoting role entails responsibility to solve all the underlying causes of a "problem". However, the demarcater is trained in handling disease, and therefore he/she is willing to take on a disease preventive role.

We are educated to take care of disease and we can do that, I think;, that is, to explain what risks worsen physical health and those which increase the risk for disease -, that is what we can do. But to try to teach people how they should live together with one another for better relationships, etc. is far beyond what I feel is my field of knowledge or what I feel I can take on...

Yes, but I wrote this with preventative care toward the most common diseases and even other conditions where prevention pays, but it doesn't only have to be from an economic viewpoint but where we have time and knowledge and can do something worthwhile, it can be sexually transmitted diseases, it can be infectious diseases of some sort...

Seeing that resources are limited, the preventive measures must be based on evidence and guidelines. The starting point for such measures, which generally are individual-oriented, is signals of physical ill-health, i.e., physiological risk factors such as high blood pressure and overweight. By modifying one's living habits, the individual is requested to take responsibility for his/her own health and thereby prevent disease from occurring.

We also have knowledge in the care sector on how one can also inform about besides medication also taking responsibility for one's health when the first sign of ill-health appears, for example, that blood pressure becomes elevated or weight becomes too much and other things that happen, that we can inform about the traditional...exercise and good food and review one's situation,...it is only the individual who can affect their own situation...

#### "The integrater"

The integrater perceives health promotion as an **extensive concept, which includes disease prevention. **Health promotion efforts, including disease prevention (in practice they are overlapping), are perceived as self-evident tasks for the entire health sector. A health promoting approach should exist side by side with the medical approach in all service processes.

It's a way of trying to integrate health promotion in the medical care. You have to work in parallel with both aspects ... You cannot just forget about one of them

The integrator is aware of the health promoting potential that lies in the everyday meetings with patients and relatives, and wishes to make the best use of this opportunity to provide the individual with "tools" for taking care of themselves.

That one actually gives self-help advice at every opportunity. Sitting in the lab chair, laying on the hospital cot and have them sitting still for five minutes to take blood pressure, yes then one has the chance.

As exemplified below, the integrater largely considers his/her ordinary task in daily work, accomplished on an individual and group level, as health promotion.

...the work we do at the antenatal clinic and the contraceptive unit is of course health promoting

Nurses at the cardiac unit rehabilitate people after infarction ... which promotes life by giving it back

In orthopedics we inform a lot about the importance of making the right moves and to use all parts of the body.

As health promoting efforts are aimed at both healthy people and people with disease, the aims may vary from prevention of disease, ill-health and injury to well-being during the final stages of life. Beside preventive measures, as illustrated below, adequate medical treatment as well as aspects of caring and rehabilitation are included. Consequently, the determinants that are targeted differ as well the measures to influence them.

If you have diabetes and come in and are prepared for insulin, if that is what you now need, then that is very health promoting.

When they are dying we see to it they are well cared for .... that they perceive well-being during the days or weeks that are left

Perhaps they have been in a respirator ... one starts by just sitting on the edge of the bed, they have zero balance for sitting. And eventually they go home supported on their own legs ... to be with from zero and until they go home and are independent ...

An important element in disease-related health promotion is facilitating recovery. Focusing on and supporting the healthy potential in all persons is important. Factors that in different ways can lead to strengthening of the body and the mind and bring meaning and zest for life are considered.

One tries to encourage them [hospitalized patients] to have visitors and perhaps go out for a little bit in the fresh air, because it can be nice to get away from here and acquire new strength in being out of the room.

Being able to master one's situation is important for people with a chronic condition in order to live as good a life as possible. Measures aimed at strengthening the patient's ability to manage his/her condition and supporting patient's trust in his/her own resources are highlighted.

When they come to us they are already sick' [heart disease], and then it is from that perspective to get them to be healthy despite that they are ill. To get them back to life, to not be sitting at home and not activating themselves, to have the courage to return to a normal life so that they don't get even sicker, surely, you are able to go out and walk, aren't you, one is saying such things all the time and it is also health promoting.

#### "The promoter"

The promoter distinguishes between primary, secondary and tertiary prevention and **views health promotion and primary prevention as "companions"**

Everything is essentially preventative if one thinks of prevention levels...Where then is the health promoting perspective? And then I think in some way that one can see the health promoting perspective and the primary prevention level as companions in this context,...foremost the primary and health promoting level is about creating...a healthy population and the best health, therefore...

From the promoter's point of view, health promotion emanates from a proactive way of thinking. The efforts shall take place before "problems" or ill-health appears. Focus is primarily on possibilities and factors that keep people healthy (salutogenesis). The perspective is creating and strengthening or empowering. In order to be successful, health promotion must be practiced on different structural levels. The aim is to create social conditions for health and to enable the individual to take control over his/her own health, as well as to maintain or improve health. The promoter considers health promotion as a matter for the health services, even though in reality the specialists in public health work reside outside the health sector.

The major specialists regarding public health, they are those who let children...trainers for clubs, it can be sports, that is, where children and youths are active...it is essentially they who are specialists, one can say, on public health.

One task for the health service, in its role as public health promoter, is to contribute knowledge about health determinants and how they can be influenced. One way of doing this is via antenatal and child welfare clinics. As living habits are believed to be established at an early stage of life, parents, expectant parents and children are in particular considered as important target groups.

I usually say that one should start with children when one talks about health, but then it hits me that it should be the parents one should start with...because that is where it starts...how children learn to live according to the family patterns at home as the foundation

Another important group pointed out by the promoter is elderly people. Proper support and help, by way of regular home visits, may facilitate maintaining health and functional capacity.

Health promoting is trying to have such a society where one can be old and live at home and maintain one's health as long as possible...that one can receive help so that one can maintain one's health and those functions one has

The great challenge for the health service is to disseminate its knowledge about health and health determinants to other groups of people. The promoter wishes to focus more on visiting activities within the local community. By participating in local community preventive efforts, for instance, by visiting schools and parent meetings, parenthood can be supported and the children encouraged taking responsibility over their own health.

I work in the local prevention group and we talk about how we are going to get youths to not use drugs, how we will strengthen the parents in the parent role so that they dare to take responsibility for their children and be parents again."

Inform at schools how extremely important it is for youths not just to sit around all day ... one can move around even if one has a geography lesson.

Additional ways of disseminating information about health and health-related issues include making use of media or participating in public debates, for instance, about alcohol policy or accommodations for elderly people. The promoter also stresses the importance of encouraging and supporting collaboration with other health actors in the local community.

And so I think, resources outside our sphere, outside the county council sphere, that's what I usually take up to empower others to think and do things for health because it already happens and that we say it is good. That one organizes exercise dancing, that one takes gardening courses, that one starts weight loss groups, etc. That this in some way is acknowledged.

In summary, it should be noted that all professional groups expressed the need for health care services to be (more actively) involved in disease prevention on individual- as well as on a population level. Our analysis resulted in three ideal types primarily aimed at capturing how they viewed their possible enrolment in health promotion efforts.

## Discussion

### Methodological considerations

To be able to mirror the range and variation in perceptions among health professionals, we included doctors, nurses, occupational therapists, counselors, physical therapists, assistant nurses and midwives in the study. We considered including psychologists and dieticians also, but because the two last discussions did not generate much new information we decided not to. Because our aim was not to compare the views of different professional groups, the over-representation of physicians and nurses was not crucial. However, it meant that great caution was taken in the analysis to also consider views from the other groups. The data collection and the analysis were performed by an interdisciplinary research team with experiences from nursing, medicine, physiotherapy, social work and staff management. This is seen as the strength of the study, which was especially important during the "peer debriefing" sessions guiding the analysis process.

### The concept of health

In this study, health professionals define health congruent with each other. None of the representatives of the seven professional groups expressed a biomedical view on health. This is surprising, because generally health services are believed to be strongly influenced by reductionist and disease-oriented views of health. Flick et al. [28] argued, for example, that there is no existing positive concept of health for the medical system that goes beyond the absence of diagnosable diseases. Fugelli [29] stated that, "While the individual experiences his/her health in connection with his/her world as a person, medicine works with parts, a molecule, an organ, a lifestyle factor." Hoffman [12], on the other hand, suggested that health care professionals apply more than one model of health depending on the context. Tibblin [30] argued in a similar way by saying that WHO's definition of health as a complete state of physical, mental and social well-being seems to be used only on special occasions, whereas most often it is used in a much more disease-oriented way. Thus, our results indicate a much better possibility for communicating about health-related issues than expected. Our informants' holistic notion of health is especially positive, because holism is essential for developing strategies for health promotion [1]. The preparation of health professionals to really address the public's health relies on an understanding of the various determinants of health [31]. This means viewing psychological, social, emotional and spiritual needs as equally important as physical needs, and that attention has to be paid to the person as a "whole" [7]. Our study illustrates that in theory, this understanding seems to be there but that this does not automatically imply that it is implemented in practical work.

It is important to interpret the agreement on the health concept in the focus group discussions as normative, i.e., indicating what a generally accepted view is. To be able to fully understand how health professionals' views on health is mirrored in their daily work, other types of more participatory research designs are needed where the concept of health is in focus [32].

### The concept of health promotion

The health professionals' descriptions of health promotion demonstrate that the concept mainly is used in a broad sense. However, the problem with such a broad interpretation is that it may contribute to confusion about what health promotion actually is and increase the risk of the concept becoming meaningless [33]. Cribb and Dines [34] asserted that it is hard to limit the boundaries of health promotion because it does not belong to any institutional setting or professional role. McQueen and Kickbusch [35] argued that the comprehensive nature of health promotion and the broad base of its practice make any short or simple definition impossible, and that the field should be left open to extensive explanations. However, this implies difficulties in creating a framework for practice. Ewles and Simnett [13] asserted that actually all measures within the health care sector strive for improving health, but they questioned whether they should be characterized as health promotion. If health promotion is a matter of enabling people to increase control over and to improve their health, all measures directed toward someone, such as removal of one's appendix, are excluded, even if they improve the health of the individual. Education in self-care or support to parents is, however, according to this view, consequently looked upon as health promotion. Our informants' various ways of associating health promotion to disease prevention demonstrate the difficulties faced in obtaining agreement about what should be incorporated in a health promoting role.

The fact that there are different opinions about the relationship between health promotion and prevention is also shown in the literature. According to some "schools", a clear distinction is more or less made, and according to others, not [6]. In practice, the term health promotion is often not clearly distinguished from disease prevention, health education or empowerment. The terms are often used complementarily or interchangeably and measures for their implementation may overlap [36].

### Health promotion in practice

Despite a shared view of health, our informants described their health promotion role very differently depending partly on how the concept of health promotion was interpreted, but also related to education, type of experiences and interests. Our data indicate that medical doctors might be more reluctant to take on a health promoting role whether working in hospital or primary care settings while other professional groups are more open to view health promotion as part of their daily practice. However to be able to compare different professional groups and health care settings further studies are required.

It should also be mentioned that if health, i.e., a sense of well-being or happiness, is only perceived as a state and not as a resource, the role of health promotion might be difficult to grasp. As one of the participants argued; "Why should the health and medical services concern themselves about this [health] ...is it because in our culture we have perceived it that it is unhealthy to be unhappy sometimes?"

The majority of the informants perceived their role in health promoting practices as meaningful and valuable, and expressed visions of developing the role, whereas some of them expressed negative attitudes towards health promotion. However, even the "demarcater", who explicitly took exception to health promotion, may to some extent operate as a health promoter (besides preventing disease), but without using the terminology. Thus, the concept of health promotion is seen as creating barriers to health promotion in itself.

Being able to recognize the nature and the capacity or potential of health promotion is crucial in order to understand, embrace and play a health promoting role.

This study shows that the meaning of health promotion must be elucidated. Within the health services there is a need to develop a multidisciplinary model for health promotion practice. Besides a clear definition of health, this model should include a definition of health promotion that clarifies the concept in relation to the following questions: Is health promotion a process, a method, a set of particular actions or an approach that encompasses a set of values? Is it about meeting the patient's needs for health care, or is it about certain outcomes? Furthermore, the model should indicate the relation between health promotion and disease prevention and exemplify what is entailed in a health promoting role. A model based on definitions and objectives that can be understood and embraced by all would be of great use in the process of re-orienting the health services in a health promoting direction.

This study is part of a larger project with the objective to develop future strategies for more health-promoting health services. Our qualitative findings raise further questions about how the views and attitudes related to health and health promotion are distributed among health professional groups with varying educational background and working experiences. This will be further explored in a forthcoming quantitative study.

## Conclusion

• This study demonstrates a shared view of health, which creates good possibilities for communicating about health-related issues among health professionals. This is vital for the prospects of developing a health promoting practice. However, there is a need to further explore how the holistic view is distributed among different health professionals within the health system.

• The concept of health promotion appears as diffuse, elusive and difficult to apply in practice. This seems to be closely linked to a lack of agreement about the relationship between health promotion and disease prevention.

• Not all of the health professionals are able to identify health promotion as part of their professional role. Yet, they may act as health promoters without using the terminology.

• The different interpretations of what constitutes health promotion can lead to unnecessary misunderstandings and pose barriers to further development of a health promoting practice. Thus, policy makers have to understand the need for further clarification of the concepts and the implications for practice. This includes a discussion about "what is in it for me"; involving health professionals who are skeptical as well as those who wish to contribute to health promotion, but have difficulties in defining their own role.

## Competing interests

The authors declare that they have no competing interests.

## Authors' contributions

The individual contributions of authors to this manuscript are; HJ, LW, ME Conception and design; HJ acquired the data; HJ, LW, ME analysed and interpreted the data; HJ drafted the article; HJ, LW, ME revised the article; HJ, LW, ME approved the final version.

## Pre-publication history

The pre-publication history for this paper can be accessed here:


